# Developmental and behavioural problems in children with severe acute malnutrition in Malawi: A cross–sectional study

**DOI:** 10.7189/jogh.07.020416

**Published:** 2017-12

**Authors:** Meta van den Heuvel, Wieger Voskuijl, Kate Chidzalo, Marko Kerac, Sijmen A Reijneveld, Robert Bandsma, Melissa Gladstone

**Affiliations:** 1 Department of Paediatrics, Hospital for Sick Children, Toronto, Ontario, Canada; 2 Department of Paediatrics, Queen Elizabeth Central Hospital, Blantyre, Malawi; 3 Global Child Health Group, Emma Children’s Hospital, Academic Medical Centre, Amsterdam, the Netherlands; 4 Department of Population Health, London School of Hygiene & Tropical Medicine, London, United Kingdom; 5 Leonard Cheshire Disability and Inclusive Development Centre, University College London, London, United Kingdom; 6 Department of Health Sciences, University Medical Center Groningen, University of Groningen, Groningen, the Netherlands; 7 Division of Gastroenterology, Hepatology and Nutrition, Hospital for Sick Children, Toronto, Ontario, Canada; 8 Centre for Global Health, Hospital for Sick Children ^,^Toronto, Ontario, Canada; 9 Department of Women and Children’s Health, University of Liverpool, Alder Hey Children's Hospital, Liverpool, United Kingdom

## Abstract

**Background:**

Early childhood development provides an important foundation for the development of human capital. Although there is a clear relation between stunting and child development outcomes, less information is available about the developmental and behavioural outcomes of children with severe acute malnutrition (SAM). Particularly an important research gap exists in Sub–Saharan Africa where there is a high prevalence of SAM and a high rate of co–occurring HIV (human immune deficiency virus) infection. Our first objective was to assess the prevalence and severity of developmental and behavioural disorders on a cohort of children admitted to an inpatient nutritional rehabilitation centre in Malawi. Our second objective was to compare the developmental and behavioural profiles of children with the two main phenotypes of SAM: kwashiorkor and marasmus.

**Methods:**

This was a cross–sectional observational study including all children hospitalized with complicated SAM in Blantyre, Malawi over an 8–month period from February to October 2015. At discharge, children were assessed with the well-validated Malawi Developmental Assessment Tool (MDAT) for gross motor, fine motor, language and social development. In children ≥24 months, emotional and behavioural problems were measured using the Strengths and Difficulties Questionnaire (SDQ).

**Results:**

150 children (55% boys) with SAM were recruited; mean age of 27.2 months (standard deviation 17.9), 27 children (18%) had pre–existing neurodisabilities (ND) and 34 (23%) had a co–occurring human immune deficiency virus (HIV) infection. All children with SAM experienced profound delays in the gross and fine motor, language and social domains. Linear regression analysis demonstrated that children with kwashiorkor scored 0.75 standard deviations lower (95% confidence interval –1.43 to –0.07) on language MDAT domain than children with marasmus when adjusted for covariates. The prosocial behaviour score of the SDQ was low in children with SAM, indicating a lack of sensitive behaviour in social interactions.

**Conclusions:**

Children with SAM have severe developmental delays after a hospital admission. Our results indicate that there might be a significant difference in developmental attainment between children with kwashiorkor and with marasmus. Future studies exploring longer–term outcomes and testing possible intervention strategies are urgently needed.

Early childhood development provides an important foundation for the development of human capital. For the first time, child development is included in the new Sustainable Development Goals (SDGs) [1]. In the recent series on child development in *The Lancet*, it is now estimated that children in Sub-Saharan Africa are the most “at-risk” for not reaching their developmental potential (66% in 2010) [2]. The first “1000 days” of life (conception to age 2) are particularly crucial for both nutrition and child development [3]. During this time, rapid growth, including brain development, places high demands on nutrition [3].

Previous literature has described the complex interactions between malnutrition, developmental delay and neurodisability, although clinically it is not always easy to explain a cause–effect relationship [4–8]. It is known that children with neurodisability (eg, cerebral palsy) have a higher risk of malnutrition [4,9]. Conversely, it is also clear that malnutrition is an important risk factor for poor child development [5,10]. In particular, there are strong associations identified between stunting (chronic malnutrition, defined by low height–for–age) and motor and cognitive development in children two years of age or younger [5,6,11,12]. Less attention has been paid to behavioural outcomes in malnourished children [13]. Scarce evidence suggests some differences in behaviour, eg, more negative affect, reduced activity, play and exploration between children who are stunted and those who are not [14].

In contrast to the amount of evidence on stunted children, developmental and behavioural outcomes in children with severe acute malnutrition (SAM) have hardly been studied. With SAM affecting some 19 million children worldwide, this is an important evidence gap [11]. The event of an acute extreme calorie shortage in children with SAM could have different implications on a child’s developmental outcome other than the chronic shortage of calories that occurs in children who are stunted [15]. Grantham–McGregor’s seminal papers did describe poor levels of development (cognitive and educational) in children post–SAM in Jamaica at the age of 2 years and further work followed up 17 of these children to 14 years [16–18]. These studies were conducted pre–HIV (human immune deficiency virus) and used different case definitions and treatment strategies for SAM thus limiting the applicability to todays’ SAM–affected populations. Only one other study has investigated developmental outcomes of children with SAM as part of an intervention study in Bangladesh. HIV status was not mentioned in this study and it is unclear how the Bayley’s Scales of Infant and Child Development (2nd version) had been adapted and validated for use in Bangladesh [19]. To our knowledge, no studies have investigated the developmental and behavioural outcomes of SAM in Sub–Saharan Africa where there is a high rate of co–occurring HIV infection in children with SAM [15,20]. “Play therapy” is one of WHO’s “10 steps” in the treatment of inpatient SAM but it is unclear how many nutritional rehabilitation centres are actually managing to implement this [21]. In order to inform policy makers and justify future developmental interventions in this population, further evidence is needed on not just the developmental but also the behavioural outcomes of SAM in this setting [22]. The primary aim of this study was to assess developmental and behavioural problems in children with SAM at time of hospital discharge in Malawi. Our first objective was to assess the prevalence and severity of developmental and behavioural disorders on a cohort of children admitted to an inpatient nutritional rehabilitation centre in Malawi. Our second objective was to compare the developmental and behavioural profiles of children with the two main phenotypes of SAM: kwashiorkor and marasmus.

## METHODS

### Study design and setting

This was a cross–sectional observational study, covering all children hospitalized for treatment of SAM in the nutrition ward of Queen Elizabeth Central Hospital (QECH), in Blantyre, Malawi, over an 8–month period from February to October 2015. QECH is a tertiary referral hospital but mainly serves as a district hospital. Children with SAM either self–present or are referred by local health category with “complicated” SAM: they have medical problems requiring inpatient care (eg, Integrated Management of Childhood Illness danger signs; pneumonia, diarrhoea); or have failed an “appetite test” (defined as the inability to eat Ready to Use Therapeutic Food) [23]. The height, weight and mid–upper–arm circumference (MUAC) of children were measured using standard WHO–based protocols and assessed using WHO 2006 child growth standards [24].

Children were defined with marasmus when they presented with MUAC<11.5cm for children less than 5 years old, or a weight–for–height z–score<–3 on the WHO growth standard [24]. Children were defined with kwashiorkor if they presented with bilateral nutritional oedema [24].

Our study included children who were participating in the “F75 trial” [ClinicalTrials.gov Identifier: NCT02246296], a randomized controlled trial of a reduced carbohydrate formulation of F75 therapeutic milk vs the traditional “F75” therapeutic milk, among children aged 6 months to 8 years with SAM. Both types of milk were only used for a short duration during the stabilisation phase and therefore would likely have no effect on the developmental outcomes. Our sample size was determined by the inclusion of children in the main study. We undertook developmental and behavioural assessments on discharge from the unit when children were clinically stable, finishing all food and had a good appetite as assessed by the clinician. Children were excluded if their parents refused to give informed consent.

### Measures

A trained and experienced research assistant (KC) with 2 years of experience with the Malawi Developmental Assessment Tool (MDAT), as well as training on the WHO UNICEF Care for Child Development Package, administered the measures of both child development and behaviour in a quiet room next to the malnutrition unit. The caregiver was present during the complete assessment. During the study period, the development assessments were independently observed by two of the authors, both paediatricians (MH, WV). In addition, after 3 months KC participated in a refresher course of the development assessment tool.

### The Malawi Development Assessment Tool (MDAT)

The MDAT is a culturally relevant developmental assessment tool that has been created for the use in African settings [25]. It examines development in the domain of gross motor, fine motor and language development through direct observation of the child as well as social development through questions to the caregiver. Cognitive items are embedded in the fine motor and language domains of the MDAT. It has 136 items (34 in each domain of development). Items are scored as “pass” or “fail” and if the child is uncooperative as “don’t know”. The MDAT has demonstrated good construct validity and sensitivity in predicting moderate to severe developmental delay in children from birth to 6 years of age and has normative values for a population of children which reflect the demographic and health surveys of the population [25]. The MDAT has good reliability in the healthy Malawian children (standardized Cronbach’s alpha 0.98 for all domains [26]) and in our SAM population without neurodisabilities (n = 121), the reliability was good for the fine motor and language domain (standardized Cronbach’s alpha 0.84 and 0.81 respectively) and acceptable for the gross motor and social domain (standardized Cronbach’s alpha: 0.76 and 0.77 respectively).

The MDAT has also demonstrated good sensitivity in detecting more subtle developmental problems in children with marasmus [25]. We calculated MDAT z–sores with the use of the MDAT reference population scores [25]. A domain z–score <–1.64 is suspect for developmental delay; this z–score identifies children who are performing worse than 68.26% of the normed population.

During the MDAT assessment, child and maternal behaviour was observed by the research assistant and reported in an observation form. This form had been adapted from the Behavior Observation Inventory from the Bayley Scales of Infant and Toddler Development [27]. It consisted of 5 items reporting about the child’s affect, engagement, anxiety and cooperativeness and about the caregiver’s involvement during the assessments. These five questions were chosen based on a previous study rating children’s behavior during development assessments in anemic children in a low–income country [28]. Since our research assistant was the only one using this observation tool, it was not translated.

### Strengths and Difficulties Questionnaire (SDQ)

The SDQ is a brief 25 item behavioural screening instrument. It is subdivided into four difficulty scales; emotional symptoms, conduct problems, inattention–hyperactivity, peer problems, and a separate fifth strength scale that enquires about the child’s behaviour in normal social interactions the “prosocial behaviour” scale [29,30]. All subscales had five questions each. An impact supplement inquires further about the existence, chronicity, and distress of problems, social and learning impairment, and burden to others [14]. Each item has to be scored on a 3–point scale with 0 = “not true”, 1 = “somewhat true” and 2 = “certainly true”. An example of a question in the inattention–hyperactivity subscale is: “Restless, overactive, cannot stay still for long”. The SDQ Total Difficulties Score (TDS) can be calculated by aggregating the scores for each difficulty scale, a higher SDQ–TDS indicates more emotional and behavioural problems. The SDQ has been used worldwide, including low– and middle–income African countries [31,32]. The SDQ has been translated into Chichewa (Malawian language), however no standardized reference values are available in Malawi (or any other African country) [33,34]. The SDQ has been validated in children older than 2 years [35]. The research assistant therefore only administered the SDQ to caregivers of children 2 years and older in our study. The internal reliability of the SDQ–TDS in our study was 0.73 (standardized Cronbach’s alpha) in children with SAM without severe neurodisabilities (n = 51), which is considered to be acceptable.

### Covariates

Neurodisability (ND) and HIV infection are common problems underlying SAM in our setting and are associated with poorer outcomes [4,15]. Children with ND will, by definition, have developmental delays in some areas and are more likely to have emotional and behavioural problems [36,37]. In our study, children were considered as having a ND if the caregiver provided a history of severe developmental delay and/or cerebral palsy at admission or this history was described in the child’s health passport.

Children with HIV have a higher risk of developmental and behavioural problems [38,39]. All children admitted with SAM were offered an HIV antibody rapid test (Abbott Laboratories, USA) as standard of care. If this first rapid test was positive, a “Uni–Gold” test (Trinity Biotech PLC, Ireland) was performed. If both tests were positive, children were considered to have an HIV infection. Baseline data about the use of co–trimoxazole prophylaxis and/or antiretroviral therapy was collected.

The following other clinically important covariates were also collected: age, sex, child disease characteristics reported during the hospital admission (severe pneumonia, diarrhoea, malaria) and family characteristics (details on the primary and secondary caregiver and parental education level).

### Analysis

We used descriptive statistics to evaluate the child’s background characteristics and the child and caregiver’s involvement in the development assessment. χ^2^ tests were used to analyse differences in child health and family characteristics between the marasmus and kwashiorkor group.

For the MDAT domains, z–scores, means and standard deviations were calculated for the entire SAM group and separately for marasmus and kwashiorkor patients. The proportion of children that had a delay in a MDAT domain (ie, had a domain score<–1.64) was computed for all groups. In addition, we examined the association between the type of SAM (marasmus or kwashiorkor) and MDAT domain z–scores with linear regression analysis. Second, we performed a linear regression analysis adjusted for clinically important covariates: gender, HIV, no education or some primary education of the primary caregiver and passive mother involvement during MDAT assessment. All children with pre–existing ND and children older than 6 years were excluded from the MDAT analysis.

Finally, we evaluated the SDQ scores in children with only SAM (SAM Only), in children with SAM and a concurrent HIV infection (SAM + HIV) and children with SAM and a pre–existing ND (SAM +ND). We used analyses of variance (ANOVA) to test for differences between these groups. All data was analysed with IBM SPSS version 20 software (www.ibm.com, New York, NY, USA).

## RESULTS

**Figure 1:**
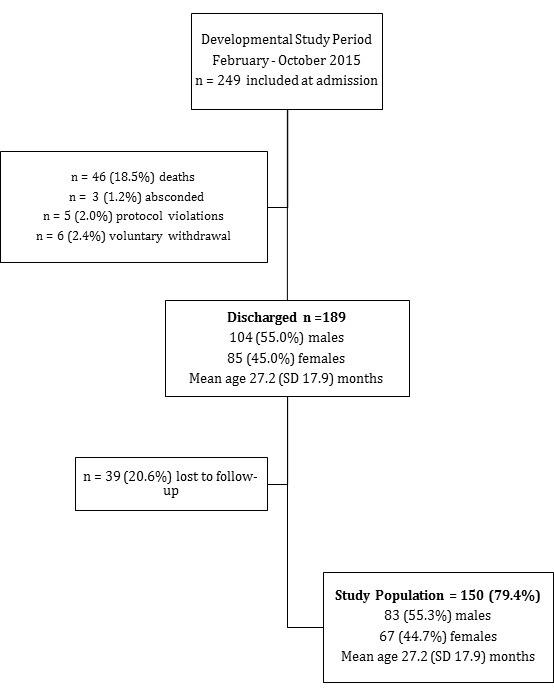
Flowchart for data collection. SD – standard deviation.

Our study period was 3 months shorter than the F75 trial. In this period a total of 249 children were included, 46 (18.5%) died and 189 were discharged to community care; 39 (20.6%) children were lost to follow–up at discharge. Reasons for loss to follow–up at discharge included that some parents wanted to leave the hospital before the developmental assessment could be done and the unavailability of our research assistant. **Figure 1** shows the flowchart for data collection. Our final study population included 150 (79.4%) children discharged after an admission with SAM to outpatient–based care with a mean age of 27.2 months (standard deviation (SD) 17.9). In our study population, 83 (55.3%) were boys, 27 (18%) had pre–existing ND and 34 (22.7%) had a concurrent HIV infection.

### Characteristics of the marasmus and kwashiorkor groups

**Table 1** shows the background characteristics according to nutritional diagnosis. Children with marasmus had a significant higher percentage of HIV (35.4% vs 12.9%, *P* = 0.001) and pre–existing ND (26.2% vs 11.8%, *P* = 0.023) compared to those with kwashiorkor (**Table 1**). There were no other statistically significant differences in child health and family characteristics between the two groups.

**Table 1:** Background characteristics of the sample by nutritional status

Characteristics	Marasmus	Kwashiorkor
**Number of children**	65 (43.3)	85 (56.7)
**Boys** (n, %)	31 (47.7)	49 (57.6)
**Age**, months (SD)	21.8 (17.2)	31.3 (17.5)
**Admission**, days (SD)	5.7 (3.0)	6.8 (3.6)
**Anthropometric measurements:**		
Weight (kg, SD)	6.3 (2.0)	9.0 (2.5)
Length (cm, SD)	71.5 (10.8)	79.1 (7.9)
MUAC (cm, SD)	10.6 (1.2)	12.4 (1.6)
Weight–for–length z–score (SD)	–3.7 (1.0)	–1.8 (1.8)
Length–for–age z–score (SD)	–3.7 (1.7)	–3.3 (1.6)
Weight–for–age z–score (SD)	–4.6 (1.1)	–3.0 (1.6)
**Health characteristics:**		
Pre–existing neurodisability (n, %)*	17 (26.2)	10 (11.8)
HIV (n, %)†	23 (35.4)	11 (12.9)
Co–trimoxazole prophylaxis (n, %)	15 (23.1)	8 (9.4)
Highly active antiretroviral therapy (n, %)	5 (7.7)	7 (8.2)
Severe pneumonia	6 (9.2)	7 (8.2)
Diarrhea	22 (33.8)	32 (37.6)
Malaria	8 (12.3)	13 (15.3)
**Family characteristics:**		
**Primary caregiver** (n, %):		
Mother	61 (93.8)	77 (90.6)
Grandparent	2 (3.1)	6 (7.1)
Other	2 (3.1)	2 (2.4)
**Highest education level** (n, %):		
No education	3 (4.6)	7 (8.2)
Some primary education	51 (78.5)	62 (72.9)
Completed primary education	3 (4.6)	1 (1.2)
Some secondary education	7 (10.8)	14 (16.5)
Completed secondary education	1 (1.5)	1 (1.0)
**Secondary caregiver** (n, %):		
Father	44 (67.7)	53 (62.4)
Grandparent	10 (15.4)	14 (16.5)
Other	8 (12.3)	13 (14.3)
No secondary caregiver	3 (4.6)	5 (5.9)
**Highest education level** (n, %):		
No education	13 (20.0)	10 (11.8)
Some primary education	20 (30.8)	39 (45.9)
Completed primary education	10 (15.4)	2 (2.4)
Some secondary education	16 (24.6)	16 (18.8)
Completed secondary education	10 (15.4)	13 (15.3)

SD – standard deviation

*χ^2^ analysis *P* = 0.023.

†χ^2^ analysis *P* = 0.001.

### Developmental outcomes

All children with SAM experienced profound delays in the gross and fine motor, language and social domains (**Table 2**). Only the language domain mean z–score in children with marasmus had a score above the cut–off score for a delay (<–1.64). Of all children with SAM, 80% had a delay in the gross motor domain, this included 41 (85%) of the children with marasmus, and 57 (78%) of the children with kwashiorkor (**Table 2**). 32 (44%) children with kwashiorkor compared with 16 (33%) of the children with marasmus were identified with a delay in all 4 MDAT domains (*P* = 0.250).

**Table 2:** MDAT z–scores and percentage suspect for delay by nutritional status*

Status	SAM (n = 121)		Marasmus (n = 48)		Kwashiorkor (n = 73)	
	Mean (SD)	Delay (n, %)	Mean (SD)	Delay (n, %)	Mean (SD)	Delay (n, %)
Gross Motor	–3.1 (1.9)	98 (79.7)	–3.0 (1.8)	41 (85.4)	–3.1 (1.8)	57 (78.1)
Fine Motor	–2.9 (1.2)	90 (73.2)	–2.8 (1.7)	37 (77.1)	–2.9 (2.6)	53 (72.6)
Language	–1.6 (1.7)	59 (48.0)	–1.2 (1.5)	20 (41.7)	–1.9 (1.8)	39 (53.4)
Social	–2.8 (2.4)	85 (69.1)	–2.2 (1.5)	31 (64.6)	–3.2 (2.8)	54 (74.0)

MDAT – Malawi Development Assessment Tool, SAM – severe acute malnutrition, SD – standard deviation

*Children with pre–existing ND (n = 27) and children older than 6 years (n = 2) were excluded from this analysis.

Linear regression analysis demonstrated that children with kwashiorkor had a significantly lower language MDAT z–score than children with marasmus (–0.75; 95% confidence interval (CI) –1.43 to –0.07), adjusted for covariates (**Table 3**). Additionally, in the unadjusted linear regression analysis children with kwashiorkor had a significantly (*P* = 0.024) lower social MDAT z–score than children with marasmus, but this effect became non–significant when we adjusted for covariates (*P* = 0.056). The other MDAT domain z–scores did not differ with statistical significance between children with marasmus and kwashiorkor (**Table 3**).

**Table 3:** Associations between nutritional status and developmental z–scores on MDAT domains: results of linear regression analyses comparing children with kwashiorkor with those with marasmus*

MDAT domain	Unadjusted B (95% CI)	*P*–value	Adjusted† B (95% CI)	*P*–value
Gross Motor	–0.07 (–0.78 to 0.65)	0.85	–0.16 (–0.94 to – 0.61)	0.68
Fine Motor	–0.22 (–1.05 to 0.61)	0.61	–0.24 (–1.14 to – 0.65)	0.59
Language	–0.76 (–1.39 to –0.14)	0.018	–0.75 (–1.43 to – 0.07)	0.032
Social	–0.99 (–1.86 to –0.13)	0.024	–0.91 (–1.84 to – 0.02)	0.056

MDAT – Malawi Development Assessment Tool CI – confidence interval

*Children with pre–existing ND (n = 27) and children older than 6 years (n = 2) were excluded from this analysis

†Adjusted for gender, HIV, no education or some primary education of the primary caregiver and passive mother involvement during MDAT assessment. B–coefficients are estimates for difference in MDAT domain z–scores in children with kwashiorkor.

Approximately a quarter of the children with SAM were not engaged: and 19% of those with kwashiorkor and 25% of those with marasmus were described as “mostly sad” during the developmental assessments. In 31% and 27% of kwashiorkor and marasmus cases respectively, mothers were described as “passively watching” and not engaged in the child’s play. There were no significant differences in the child’s affect (*P* = 0.210) engagement (*P* = 0.670), anxiety (*P* = 0.600) and cooperativeness (*P* = 0.740) of the child and in the caregiver’s involvement (*P* = 0.370) between the marasmus and kwashiorkor groups (**Table 4**).

**Table 4:** Mother and child cooperativeness during MDAT assessment, by nutritional status (No, %)

Assessment	Affect of child	Engagement	Cooperativeness		Fear		Mother involvement	
	**Mostly sad**	**Uninterested**		**Very difficult**		**Too anxious**		**Passively watched**	
Marasmus	12 (25.0)	13 (27.1)	13 (27.1)		5 (10.4)		13 (27.1)	
Kwashiorkor	14 (18.7)	20 (26.7)	20 (26.7)		5 (6.7)		23 (30.7)	

### Behavioural outcomes

The mean (SD) SDQ–TDS scores were 10.1 (4.2), 12.2 (5.5) and 15.5 (3.8) in the children with SAM Only, SAM+HIV and SAM + ND respectively. **Table 5** describes the SDQ Total Difficulties scores and the scores on the different subscales of the SDQ. The children with SAM+ND had a significantly higher SDQ–TDS score than the children with SAM Only (*P* < 0.001) or children with SAM+HIV (*P* = 0.048). We found no statistically significant differences in SDQ–TDS scores between the children with SAM Only and those with an HIV infection (*P* = 0.130). No calculations were done to examine the different subdomains between the different groups because of the small sample size.

**Table 5:** Background characteristics and Strengths and Difficulties Questionnaire scores by SAM and HIV status in children ≥24 month–old

	SAM only* (n = 37)	SAM + HIV† (n = 14)	SAM + ND‡ (n = 15)
**Group characteristics:**			
Kwashiorkor (n, %)	33 (89.2)	8 (57.1)	9 (60.0)
Male (n, %)	20 (54.1)	8 (57.1)	11 (73.3)
Age in months (mean, SD)	37.6 (15.7)	42.7 (22.5)	50.8 (15.4)
**Strengths and Difficulties Questionnaire** (mean, SD):			
Emotional problems	2.1 (1.4)	3.1 (2.6)	2.6 (1.5)
Conduct problems	1.9 (1.5)	2.6 (1.8)	3.7 (1.5)
Hyperactivity–inattention problems	4.1 (1.5)	4.6 (1.0)	4.5 (0.9)
Peer problems	1.9 (1.5)	1.9 (1.7)	4.7 (1.6)
Prosocial behavior	3.2 (2.0)	3.2 (2.8)	0.1 (0.4)
Total Difficulties Score§	10.1 (4.2)	12.2 (5.5)	15.5 (3.8)
**Impact supplement:**			
Overall difficulties in emotions, concentration, behavior or being able to get on with other people? (n,%)	5 (13.5)	3 (21.4)	14 (93.3)

SAM – severe acute malnutrition, HIV – human infectious virus, SD – standard deviation, ND – neurodisability

*SAM only: 3 children missing.

†SAM + HIV group: children with both SAM and HIV: 1 child missing

‡SAM + ND group: children with both SAM and a pre–existing neurodisability: 2 children also were HIV positive.

§ANOVA analysis demonstrated a significant difference between the SAM only and SAM+ ND group (*P* < 0.001) and between the SAM+HIV and SAM+CP group (*P* = 0.048).

## DISCUSSION

We report on the severe developmental delays present in a well–characterized clinical population of children admitted with SAM to a nutrition rehabilitation unit in an African setting in a first study on this topic for decades. We have confirmed previous findings showing that SAM is associated with severe developmental delay. Moreover, our results indicate that there might be a significant difference in developmental attainment between children with kwashiorkor and with marasmus. Our study is unique in adding to the very sparse literature describing child behaviour as well as child development in young children with SAM.

In our study, children with kwashiorkor had a significantly worse language delay compared to children with marasmus at discharge. One explanation for this could be a difference in neurological involvement between kwashiorkor and marasmus. The striking neurological irritability of children with kwashiorkor has been identified as an important clinical feature [40]. However a recent case–report series did not reveal a difference in cerebral MRI findings between children with marasmus and kwashiorkor [41]. Secondly, the difference in language delay could be explained by a difference in the social environment between children with kwashiorkor and marasmus. Rytter et al. described lower breastfeeding rates in children with kwashiorkor, which might be related to decreased maternal care and stimulation [42]. Alternatively, the difference in language delay could be related to the differences in pathophysiology between kwashiorkor and marasmus. Recent evidence has linked the gut microbiome as being a causative factor in kwashiorkor [43]. Some have proposed that the immaturity of the gut microbiota (as seen in kwashiorkor) leads to the lack of production of important neurotransmitters and agents which are linked to brain development in children such as insulin–like growth factor (IGF–1) [44]. However studies comparing the microbiome of children with kwashiorkor and marasmus are still lacking.

Our study examined the child’s social – emotional skills using direct observation (rated during the MDAT assessment) and with the use of two different questionnaires (MDAT social domain, SDQ). When examining the behaviour outcomes of children with SAM, we found different SDQ scores than previously reported in a World Bank evaluation of Malawian preschool (n = 1815) children attending community based childcare centres [34]. In the preschool group, the mean (SD) SDQ TDS was 13.9 (5.0) and the mean scores (SD) for the domains were: emotional problems (3.7 ± 2.3), conduct problems (3.0 ± 2.2), hyperactivity/inattention problems (4.5 ± 2.0), peer problems (2.6 ± 1.8) and the prosocial subscale (6.0 ± 2.4). Surprisingly, the mean SDQ TDS in the preschool children was higher than the mean SDQ TDS of the children in our study with SAM and SAM and HIV. In addition, the prosocial behaviour score was very low (3.2 ± 2.0) in children with SAM compared to the preschool children (6.0 ± 2.4), indicating a lack of sensitive behaviour in social interactions in our study population. An explanation might be that the children in our study did not demonstrate a high level of problematic behaviour because they were subdued and not very interactive indicated by the high level of children that were observed to be “not engaged” and “mostly sad” during our behaviour observation and the low MDAT social z–scores. The SDQ might not be the best scale to assess behavioural difficulties in children with SAM at discharge. Unfortunately our sample size was to small to investigate any differences in emotional and behavioural problems between children with kwashiorkor and marasmus. Additionally, our sample size might also have been too small to identify differences in our observation scale between marasmus and kwashiorkor.

### Limitations of this study

Limitations of our study include its cross–sectional design and the assessment of developmental and problems only at discharge from the ward. We did not have any previous details about the development of the child before admission and no details about the home environment. Our findings regarding a significant difference in language delay between the children in the marasmus and kwashiorkor group might also have been explained by the age difference between both groups. The kwashiorkor children were significantly older although the MDAT z–scores are adjusted for age. Unfortunately, our sample size was determined by the main trial so for an age–stratified analysis our sample size was too small.

It is likely that some developmental domains will continue to improve during the final few weeks of nutritional rehabilitation in the community [45]. This is especially true since WHO guidelines do not encourage using target–weight as hospital discharge criteria and admission to hospital is much shorter than it was a decade ago [21]. Despite clinical stabilization and improved appetite, children with SAM are still very brittle on discharge.

Our study has utilized a well–validated African developmental assessment tool, the MDAT, as an outcome measure however behaviour was measured using the SDQ, which has only been translated and not validated in Malawi. There are currently no SDQ cut–off scores available for Malawi, so it is unclear how many children with complicated SAM had SDQ scores in the clinical range. This may have led to a lack of significant results from this perspective. The five questions in the observation of child and maternal behaviour were not validated in this study and could have been confounded by how well the child performed on the MDAT test. Because our sample size was lower for children with marasmus, our power estimate was probably <0.80 to correctly identify a difference between children with marasmus and kwashiorkor.

Finally, we assessed only children with complicated SAM: these are by definition the most vulnerable and findings should not be directly extrapolated to the much wider group of children with uncomplicated SAM who are treated as outpatients under current protocols [46].

## CONCLUSIONS

Findings from this study demonstrated that children with SAM have severe developmental delays after a hospital admission. Future research should focus on the longer–term developmental and behavioural follow–up outcomes of children with SAM in a Sub–Saharan African setting. These results demonstrate the need for developmental interventions during treatment of SAM in a hospital or community setting. Both McGregor’s and Nahar’s study identified significant differences in child development outcomes between children with SAM who received an intervention and children who did not. However, both studies were not randomized and had other limitations for example a high level of selection bias and attrition bias [47]. Therefore there is a high need for randomized intervention studies testing the efficacy, effectiveness and cost–effectiveness of various developmental support packages [48]. Our study identified a high proportion of children with pre–existing neurodisabilities (18%), future research should also investigate psychosocial interventions specifically targeted for these children. Developmental interventions need larger investments in staff and training to ensure that they are implemented and maintained in the many centres which see children with SAM to allow for the full development of these vulnerable children in order to improve future human capital and potential.

## References

[1] United Nations. Open working group proposal for sustainable development. Available: https://sustainabledevelopment.un.org/content/documents/1579SDGs Proposal.pdf. Accessed: 27 October 2016.

[2] Black MM, Walker SP, Fernald LC, Andersen CT, DiGirolamo AM, Lu C, Lancet Early Childhood Development Series Steering Committee (2017). Early childhood development coming of age: Science through the life course.. Lancet.

[3] Black MM, Walker SP, Wachs TD, Ulkuer N, Gardner JM, Grantham-McGregor S (2008). Policies to reduce undernutrition include child development.. Lancet.

[4] Gladstone M, Mallewa M, Alusine Jalloh A, Voskuijl W, Postels D, Groce N (2014). Assessment of neurodisability and malnutrition in children in Africa.. Semin Pediatr Neurol.

[5] Sudfeld CR, McCoy DC, Danaei G, Fink G, Ezzati M, Andrews KG (2015). Linear growth and child development in low- and middle-income countries: a meta-analysis.. Pediatrics.

[6] Grantham-McGregor S, Cheung YB, Cueto S, Glewwe P, Richter L, Strupp B, International Child Development Steering Group (2007). Developmental potential in the first 5 years for children in developing countries.. Lancet.

[7] Brown JL, Pollitt E (1996). Malnutrition, poverty, and intellectual development.. Sci Am.

[8] Kerac M, Postels DG, Mallewa M, Alusine Jalloh A, Voskuijl WP, Groce N (2014). The interaction of malnutrition and neurologic disability in Africa.. Semin Pediatr Neurol.

[9] Kuperminc MN, Stevenson RD (2008). Growth and nutrition disorders in children with cerebral palsy.. Dev Disabil Res Rev.

[10] Groce N, Challenger E, Berman-Bieler R, Farkas A, Yilmaz N, Schultink W (2014). Malnutrition and disability: Unexplored opportunities for collaboration.. Paediatr Int Child Health.

[11] Black RE, Victora CG, Walker SP, Bhutta ZA, Christian P, de Onis M, Maternal and Child Nutrition Study Group (2013). Maternal and child undernutrition and overweight in low-income and middle-income countries.. Lancet.

[12] Walker SP, Wachs TD, Grantham-McGregor S, Black MM, Nelson CA, Huffman SL (2011). Inequality in early childhood: Risk and protective factors for early child development.. Lancet.

[13] Wachs TD (1995). Relation of mild-to-moderate malnutrition to human development: correlational studies.. J Nutr.

[14] Gardner JM, Grantham-McGregor S, Himes J, Chang S (1999). Behaviour and development of stunted and nonstunted Jamaican children.. J Child Psychol Psychiatry.

[15] Lelijveld N, Seal A, Wells J, Kirkby J, Opondo C, Chimwezi E (2016). Chronic disease outcomes after severe acute malnutrition in Malawian children (ChroSAM): a cohort study.. Lancet Glob Health.

[16] Grantham-McGregor S, Powell C, Walker S, Chang S, Fletcher P (1994). The long-term follow-up of severely malnourished children who participated in an intervention program.. Child Dev.

[17] Grantham-McGregor S, Schofield W, Harris L (1983). Effect of psychosocial stimulation on mental development of severely malnourished children: An interim report.. Pediatrics.

[18] Grantham-McGregor S, Stewart M, Powell C (1991). Behaviour of severely malnourished children in a Jamaican hospital.. Dev Med Child Neurol.

[19] Nahar B, Hamadani JD, Ahmed T, Tofail F, Rahman A, Huda SN (2009). Effects of psychosocial stimulation on growth and development of severely malnourished children in a nutrition unit in Bangladesh.. Eur J Clin Nutr.

[20] Heikens GT, Bunn J, Amadi B, Manary M, Chhagan M, Berkley JA, Blantyre Working Group (2008). Case management of HIV-infected severely malnourished children: Challenges in the area of highest prevalence.. Lancet.

[21] Ashworth A, Khanum S, Jackson ASC. Guidelines for the inpatient treatment of severely malnourished children. World Health Organization; Geneva; 2003.

[22] Grantham-McGregor SM, Fernald LC, Kagawa RM, Walker S (2014). Effects of integrated child development and nutrition interventions on child development and nutritional status.. Ann N Y Acad Sci.

[23] Trehan I, Manary MJ (2015). Management of severe acute malnutrition in low-income and middle-income countries.. Arch Dis Child.

[24] World Health Organization, United Nations Children’s Fund. WHO Child growth standards and the identification of severe acute malnutrition in infants and children. Available: http://www.who.int/nutrition/publications/severemalnutrition/9789241598163/en/. Accessed: 30 July 2014. 24809116

[25] Gladstone M, Lancaster GA, Umar E, Nyirenda M, Kayira E, van den Broek NR (2010). The Malawi developmental assessment tool (MDAT): The creation, validation, and reliability of a tool to assess child development in rural African settings.. PLoS Med.

[26] Gladstone M. Correspondence with the author.

[27] Bayley N. Bayley Scales of Infant Development III. San Antonio: Psychological Corporation; 2005.

[28] Tofail F, Hamadani JD, Mehrin F, Ridout DA, Huda SN, Grantham-McGregor SM (2013). Psychosocial stimulation benefits development in nonanemic children but not in anemic, iron-deficient children.. J Nutr.

[29] Goodman R (2001). Psychometric properties of the strengths and difficulties questionnaire.. J Am Acad Child Adolesc Psychiatry.

[30] Stone LL, Otten R, Engels RC, Vermulst AA, Janssens JM (2010). Psychometric properties of the parent and teacher versions of the strengths and difficulties questionnaire for 4- to 12-year-olds: A review.. Clin Child Fam Psychol Rev.

[31] Cluver L, Gardner F (2006). The psychological well-being of children orphaned by AIDS in Cape Town, South Africa.. Ann Gen Psychiatry.

[32] Menon A, Glazebrook C, Campain N, Ngoma M (2007). Mental health and disclosure of HIV status in Zambian adolescents with HIV infection: Implications for peer-support programs.. J Acquir Immune Defic Syndr.

[33] Kariger P, McConnell C, Mwera J, Durazo J, Chulu B, Kholowa F, et al. Chichewa version Strengths and Difficulties Questionnaire. Available: http://www.sdqinfo.com/py/sdqinfo/b3.py?language=Chichewa. Accessed: 23 October 2013.

[34] Durazo J, Fernald L, Kariger P, Kholowa F, McConnell C, McDonald CN, et al. Protecting Early Childhood Development in Malawi: Baseline Report. Washinginton DC; World Bank: 2015.

[35] Theunissen MH, Vogels AG, de Wolff MS, Reijneveld SA (2013). Characteristics of the Strengths and Difficulties questionnaire in preschool children.. Pediatrics.

[36] Rackauskaite G, Bilenberg N, Bech BH, Uldall P, Ostergaard JR (2016). Screening for psychopathology in a national cohort of 8- to 15-year-old children with cerebral palsy.. Res Dev Disabil.

[37] Morris C, Janssens A, Tomlinson R, Williams J, Logan S (2013). Towards a definition of neurodisability: A delphi survey.. Dev Med Child Neurol.

[38] Abubakar A, Van Baar A, Van de Vijver FJ, Holding P, Newton CR (2008). Paediatric HIV and neurodevelopment in Sub-Saharan Africa: a systematic review.. Trop Med Int Health.

[39] Boivin MJ, Bangirana P, Nakasujja N, Page CF, Shohet C, Givon D (2013). A year-long caregiver training program improves cognition in preschool Ugandan children with human immunodeficiency virus.. J Pediatr.

[40] Williams CD (1933). A nutritional disease of childhood associated with a maize diet.. Arch Dis Child.

[41] Hazin AN, Alves JG, Rodrigues Falbo A (2007). The myelination process in severely malnourished children: MRI findings.. Int J Neurosci.

[42] Rytter MJ, Namusoke H, Babirekere-Iriso E, Kaestel P, Girma T, Christensen VB (2015). Social, dietary and clinical correlates of oedema in children with severe acute malnutrition: A cross-sectional study.. BMC Pediatr.

[43] Smith MI, Yatsunenko T, Manary MJ, Trehan I, Mkakosya R, Cheng J (2013). Gut microbiomes of Malawian twin pairs discordant for kwashiorkor.. Science.

[44] Goyal MS, Venkatesh S, Milbrandt J, Gordon JI, Raichle ME (2015). Feeding the brain and nurturing the mind: Linking nutrition and the gut microbiota to brain development.. Proc Natl Acad Sci U S A.

[45] Faurholt-Jepsen D, Hansen KB, van Hees VT, Christensen LB, Girma T, Friis H (2014). Children treated for severe acute malnutrition experience a rapid increase in physical activity a few days after admission.. J Pediatr.

[46] World Health Organization. Updates on the management of severe acute malnutrition in infants and children. Available: http://www.who.int/nutrition/publications/guidelines/updates_management_SAM_infantandchildren/en/index.html. Accessed: 20 Jun 2016. 24649519

[47] Daniel AI, Bandsma RH, Lytvyn L, Voskuijl WP, Potani I, van den Heuvel M (2017). Psychosocial stimulation interventions for children with severe acute malnutrition: A systematic review.. J Glob Health.

[48] Olusanya BO (2011). Priorities for early childhood development in low-income countries.. J Dev Behav Pediatr.

